# Microbial Poly-γ-Glutamic Acid (γ-PGA) as an Effective Tooth Enamel Protectant

**DOI:** 10.3390/polym14142937

**Published:** 2022-07-20

**Authors:** Mattia Parati, Louisa Clarke, Paul Anderson, Robert Hill, Ibrahim Khalil, Fideline Tchuenbou-Magaia, Michele S. Stanley, Donal McGee, Barbara Mendrek, Marek Kowalczuk, Iza Radecka

**Affiliations:** 1 Faculty of Science and Engineering, University of Wolverhampton, Wolverhampton WV1 1 LY, UK; ibrahim.khalil2@wlv.ac.uk (I.K.); f.tchuenbou-magaia@wlv.ac.uk (F.T.-M.); 2 Institute of Dentistry, Barts and The London School of Medicine and Dentistry, Queen Mary University of London, London E1 2AD, UK; louisaclarke35@hotmail.co.uk (L.C.); r.hill@qmul.ac.uk (R.H.); 3 Scottish Association for Marine Science, Scottish Marine Institute, Oban PA37 1QA, UK; michele.stanley@sams.ac.uk; 4 AlgaeCytes Limited, Discovery Park House, Ramsgate Road, Sandwich, Kent CT13 9ND, UK; donalmcgee@algaecytes.com; 5 Centre of Polymer and Carbon Materials, Polish Academy of Sciences, M. Curie-Sklodowskiej 34, 41-819 Zabrze, Poland; bmendrek@cmpw-pan.edu.pl (B.M.); marek.kowalczuk@cmpw-pan.edu.pl (M.K.)

**Keywords:** γ-PGA, oral biology, biotechnology, *Bacillus*, statherin, delivery systems, polymer engineering

## Abstract

Poly-γ-glutamic acid (γ-PGA) is a bio-derived water-soluble, edible, non-immunogenic nylon-like polymer with the biochemical characteristics of a polypeptide. This *Bacillus-*derived material has great potential for a wide range of applications, from bioremediation to tunable drug delivery systems. In the context of oral care, γ-PGA holds great promise in enamel demineralisation prevention. The salivary protein statherin has previously been shown to protect tooth enamel from acid dissolution and act as a reservoir for free calcium ions within oral cavities. Its superb enamel-binding capacity is attributed to the L-glutamic acid residues of this 5380 Da protein. In this study, γ-PGA was successfully synthesised from *Bacillus subtilis* natto cultivated on supplemented algae media and standard commercial media. The polymers obtained were tested for their potential to inhibit demineralisation of hydroxyapatite (HAp) when exposed to caries simulating acidic conditions. Formulations presenting 0.1, 0.25, 0.5, 0.75, 1, 2, 3 and 4% (*w*/*v*) γ-PGA concentration were assessed to determine the optimal conditions. Our data suggests that both the concentration and the molar mass of the γ-PGA were significant in enamel protection (*p* = 0.028 and *p* < 0.01 respectively). Ion Selective Electrode, combined with Fourier Transform Infra-Red studies, were employed to quantify enamel protection capacity of γ-PGA. All concentrations tested showed an inhibitory effect on the dissolution rate of calcium ions from hydroxyapatite, with 1% (wt) and 2% (wt) concentrations being the most effective. The impact of the average molar mass (M) on enamel dissolution was also investigated by employing commercial 66 kDa, 166 kDa, 440 kDa and 520 kDa γ-PGA fractions. All γ-PGA solutions adhered to the surface of HAp with evidence that this remained after 60 min of continuous acidic challenge. Inductively Coupled Plasma analysis showed a significant abundance of calcium ions associated with γ-PGA, which suggests that this material could also act as a responsive calcium delivery system. We have concluded that all γ-PGA samples tested (commercial and algae derived) display enamel protection capacity regardless of their concentration or average molar mass. However, we believe that γ-PGA D/L ratios might affect the binding more than its molar mass.

## 1. Introduction

Over the years, the oral health care industry has attempted to develop therapeutic oral hygiene products to alleviate the symptoms of xerostomia and maintain the integrity of the dental hard tissues within the oral cavity [1,2]. Xerostomia is prolonged dry mouth, a condition which occurs as a result of decreased salivary flow (below 40–50% of its average value) [1,3]. Although there are many causes of xerostomia; the use of multiple medicines in the aging population is one of the most common causes of this condition [4]. Xerostomia can also arise following radiotherapy treatment, especially for head and neck cancers; following the development of atrophic glandular tissue [5]. It is well established that following radiotherapy, caries progress rapidly as a result of the loss of protection from saliva and alterations in enamel composition with loss of calcium and phosphorous ions [5]. In addition, autoimmune conditions (e.g., Sjogren’s syndrome) can also cause xerostomia [4]. The incidence of xerostomia is estimated to be 22% [6]. Although there are commercial products available to ease the symptoms derived from xerostomia, none of them are capable of dealing with dental enamel demineralisation [7].

Saliva plays a fundamental role in oral health. Saliva is the transparent biofluid secreted into the mouth via the ductal systems associated with predominately three pairs of major salivary glands; with approximately 99% of it being water. The remaining 1% is proteins, mineral ions, electrolytes, immunoglobulins, and large mucopolysaccharides [8]. Saliva exhibits specific and non-specific physico-chemical properties and it is essential for lubrication, cleansing of the oral cavity, antimicrobial and antiviral activity, wound healing, buffering of acids, and supersaturated concentrations of calcium and phosphate ions, which are essentially the mineral calcium hydroxyapatite (HAp) required for the protection of dental hard tissues [9]. The rheological properties displayed by saliva are provided by mucopolysaccharide fraction of saliva and not by statherin or other proteins [10].

A fundamental role of saliva is its association in the formation of the acquired enamel pellicle (AEP) [11]. The AEP is formed by the adsorption of four salivary phosphoproteins, the first being statherin, in sequence onto the enamel surface, protecting the enamel from acid dissolution and acting as a reservoir for free calcium ions adjacent to the surface [12]. The mode of action of statherin is related to the high hydroxyapatite binding propensity of glutamic acid residues near its N-terminus [12,13].

The mineral in the enamel is a calcium-deficient carbonated hydroxyapatite (Ca_10_(PO_4_)_6_OH_2_) with some extrinsically sourced fluoride. From a chemical point of view, the solubility isotherm for HAp, calculated from the solubility of calcium hydroxyapatite, shows that when the pH of the surrounding solution is less than the “critical pH” (i.e., the pH at which a solution is just saturated with the enamel mineral) the solution is undersaturated and therefore demineralisation will occur [14]. These conditions can be simulated in in vitro models using cariogenic or erosiogeneic simulating artificial demineralisation solutions. Clinically, erosion (and caries) of enamel occurs when the pH of the surrounding solution in the oral cavity is below the critical pH at normal salivary calcium concentrations [15]. Statherin prevents such mechanisms by effectively shielding the enamel layer and thus, modifying, the effective solubility of calcium hydroxyapatite, reducing the interaction between the low pH, calcium and phosphate deficient solution and the hydroxyapatite layer [12,13].

An unfortunate and costly consequence of xerostomia is severe dental hard tissue destruction, leading to a clinical condition termed “rampant dental caries” [16]. This condition can render the patient edentulous, requiring significant dental intervention [16]. Therefore, investigation into new biopolymeric materials, such as poly(d/l- γ-glutamic acid), could provide novel solutions to tackle these issues.

**Figure 1:**
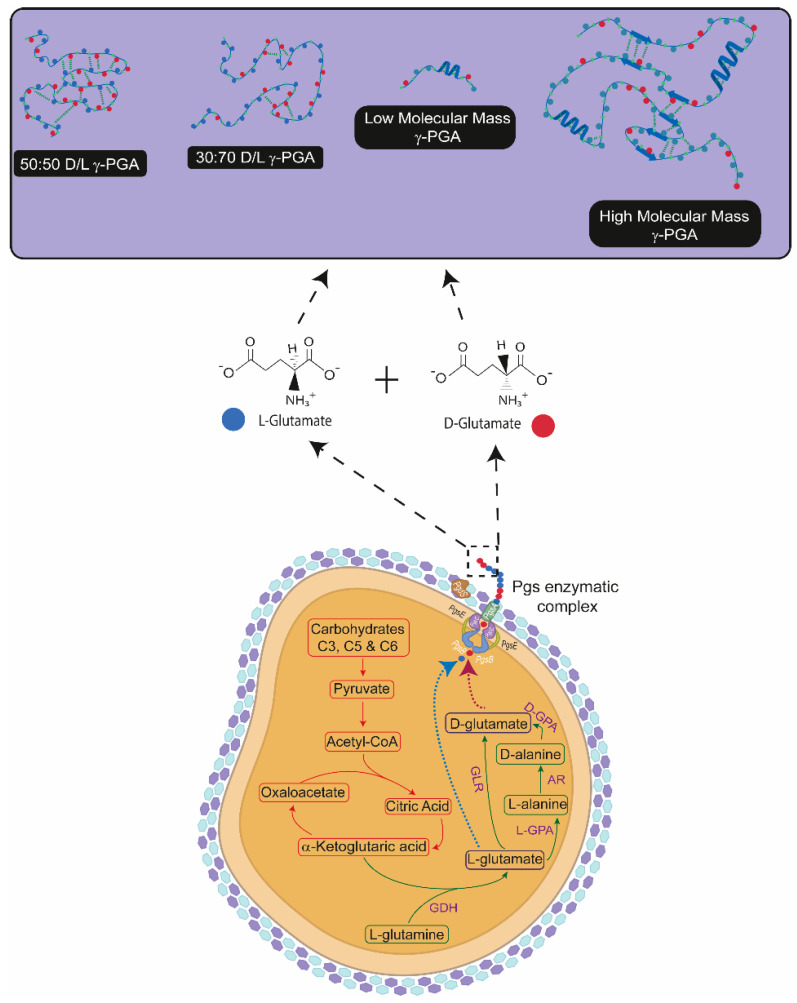
Factors and pathways involved in the biosynthesis of poly (γ-glutamic acid) (γ-PGA). Herein, the metabolic pathway (in red) of γ-PGA producers has been summarised. In red, the energy generation pathways have been shown. Similarly, γ-PGA generation intermediates/components have been presented in green. The figure also illustrates polymerisation of d- and l-glutamic acid monomers (red and blue dots respectively) into a polymeric chain by the enzymatic complex. The physical arrangement of the polymer, with varying D/L ratios, in solution has also been summarised. Light blue and purple hexagons represent cross-linked NAM and NAG moieties of Gram-positive peptidoglycan. GDH—Glutamate dehydrogenase; L-GPA—l-glutamic acid:pyruvate aminotransferase; AR—alanine racemase; D-GPA—d-glutamic acid:pyruvate aminotransferase; GLR—glutamate racemase.

Poly(d/l-γ-glutamic acid) is an extracellularly secreted protein-like polymeric material synthesised by an array of Prokaryotic and Eukaryotic organisms [17,18]. In contrast to other polypeptides, the biosynthesis of γ-PGA occurs through an enzymatic complex which is responsible for the polymerisation of both d- and l-glutamic acid monomers (see Figure 1). By employing an enzymatic complex, producers can greatly change the properties of the polymer in response to changing environmental conditions [19].

Both the biochemical properties and physical properties of γ-PGA suggest its suitability as an anti-cariogenic agent, as well as a therapeutic treatment to prevent rampant caries associated with xerostomia [12]. It has been shown that γ-PGA has saliva-like viscosity and calcium ion binding capability and shows great similarity to statherin, which hints at its suitability for xerostomia treatment [20]. Further, it has been shown that γ-PGA can increase salivary flow, and gamma-peptidic bonds help γ-PGA to prevent degradation by microbial proteases [17,18,20,21]. In addition, the presence of carboxylic acid-rich pendant groups (Figure 1) helps γ-PGA to ionically interact with protons and other charged molecules/compounds [20]. In this study, we propose the supplementation of such material within acidic drinks, to decrease the overall enamel demineralisation which occurs a great deal from the ever-increasing consumption of such beverages, or its supplementation through other therapeutic delivery systems.

Given that the biosynthesis of γ-PGA is regulated by enzymatic activity (Figure 1), produced polymers can vary in molar mass, D/L ratio and chain association with salts [22,23]. Each one can have a significant impact on chain physical arrangement, and ultimately on polymeric behaviour [24]. In nature, variation of polymeric chemical structure helps microorganisms survive in challenging conditions, including nutrient shortage [25,26], high salt and/or heavy metal concentrations [27,28,29].

The biosynthesis of γ-PGA is greatly dependent on the strain, cultivation time, cultivation conditions and substrate. The fermentation system, mode and conditions also play an important role in determining polymeric yields and characteristics [18]. The understanding of these, and how they are further altered by the substrate and strain, is vital in obtaining the maximum amounts of specific polymeric composites in a consistent manner [30]. Another great barrier towards the large-scale commercialisation of γ-PGA is the cost of production. Currently, expensive standard media components are employed for the synthesis of this biopolymer [31,32]. To our knowledge, no complex waste media is currently being used for commercial synthesis of this valuable biopolymer. However, with the rapid utilisation of available land for human activities, relatively unexplored oceans are gaining attention as a source for food or novel biomaterials [33,34]. As a result of this activity, non-edible, nutrient-rich seaweed and algae industrial by-products are created, which could potentially be utilised as substrates for cost-effective biosynthesis of γ-PGA [32,34,35]. Herein, we attempted to synthesise γ-PGA using both micro- and macro-algae complex media. Further, as production methods are developed to deliver cost-effective γ-PGA, here we assess whether γ-PGA synthesised from complex waste media, could protect the enamel from acid dissolution and, therefore, reduce the overall enamel demineralisation in patients presenting xerostomia. 

## 2. Materials and Methods

### 2.1. Biosynthesis of γ-PGA

#### 2.1.1. Microorganism

*Bacillus subtilis* natto (ATCC15245) obtained from the National Collection of Industrial and Marine Bacteria (NCIMB) were freeze-dried and kept at −20 °C. Before use, cultures were resuscitated and grown on Tryptone Soya Agar TSA (Lab M, Heywood, UK) overnight at 37 °C. Highly mucoid colonies were selected and grown aerobically in shake flasks containing 100 mL of TSB medium (Lab M, Heywood, UK) at 37 °C for 24 h. 

#### 2.1.2. Fermentation Media

Standard GS Medium (50 g/L NaCl) was composed of 20 g/L l-glutamate purchased from Fischer Chemicals Ltd. (Loughborough, UK), 50 g/L sucrose, 2.7 g/L KH_2_PO_4_, 4.2 g/L Na_2_HPO_4_, 50 g/L NaCl, 5 g/L MgSO_4_ ·7H_2_O, and 1 mL/L of Murashige-Skoog vitamin solution, all purchased from Sigma-Aldrich (Irvine, UK). The pH of this medium was adjusted to 6.8 using NaOH purchased from Fischer Chemicals Ltd. (Loughborough, UK). 

Modified GS Medium (0 g/L NaCl) was composed of 20 g/L l-glutamate purchased from Fischer Chemicals Ltd. (Loughborough, UK), 50 g/L sucrose, 2.7 g/L KH_2_PO_4_, 4.2 g/L Na_2_HPO_4_, 5 g/L MgSO_4_ ·7H_2_O, and 1 mL/L of Murashige-Skoog vitamin solution, purchased from Sigma-Aldrich (Irvine, UK). The pH of this medium was adjusted to 6.8 using NaOH purchased from Fischer Chemicals Ltd. (Loughborough, UK). 

Macroalgal medium was composed of 40 g/L *Laminaria digitata* flakes (Cornish Seaweed Ltd., Helston, UK) or *Laminaria digitata* powder (Scottish Association of Marine Science, Oban, Scotland, UK), and 10 g/L l-glutamate (Fischer Chemicals Ltd., Loughborough, UK), 25 g/L Sucrose (Sigma-Aldrich, Irvine, UK). The media was pre-treated with the aid of high shear mixing (Silverson, East Longmeadow, MA, USA) at 3000 rpm for 3 min. *Laminaria digitata*, was cultivated at the Port-a-Bhuiltin seaweed farm, operated by the Scottish Association for Marine Science (SAMS, Oban, Scotland, UK). Appropriate thalli of *Laminaria digitata* were hand-harvested using knives to cut the stipe above the blade, and placed into clean sampling containers (60 L plastic boxes). Samples were collected between 7 April 2021 and 1 July 2021. Seaweed thalli were macerated and homogenised using an industrial-sized mincer (Hobart, Model E4522) and frozen immediately at −20 °C, freeze dried and the dried material milled to <1 mm in size using a coffee grinder.

Microalgal medium was composed of 40 g/L *Nanochloropsis oceanica* (Algaecytes Ltd., Discovery Park, Kent, UK) previously ethanol extracted (Tennants Distribution, Manchester, UK), 10 g/L L-glutamate (Fischer Chemicals Ltd., Loughborough, UK), and 25 g/L Sucrose (Sigma-Aldrich, Irvine, UK). The post-oil extracted spent microalgal biomass was provided courtesy of AlgaeCytes Limited (Algaecytes Ltd., Discovery Park, Kent, UK). To generate the spent biomass, the spray dried *Nanochloropsis oceanica* biomass underwent AlgaeCytes’ in-house omega-3 oil extraction process to generate their AVEPA^TM^ range of eicosapentaenoic acid (C20:5_(n−3)_, EPA) vegan friendly enriched algal oil. The post-oil extracted biomass was transferred to a vacuum oven for 18 h to vent off residual ethanol solvent. The proximate biochemical composition of the spent biomass was determined using FT-IR analysis [36]; comprising of approximately 3.7% DW lipids, 13.7% DW protein and 18.0% carbohydrates.

All media were prepared using deionised water and sterilised by autoclaving at 121 °C for 15 min. The sucrose solution was sterilised separately and vitamin solution was filter sterilised (0.22 µm, Fischer Chemicals Ltd., Loughborough, UK) and added separately to the medium. 

#### 2.1.3. Cultivation Parameters

Batch cultures were carried out in 250 mL shake flasks. The cultures were inoculated by thawing the frozen cells in a 37 °C bath (Grant Instruments, Fischer Chemicals Ltd., Loughborough, UK) and 5% (*v*/*v*) of inoculum was added to the cultivation media. Growth temperature was kept at 37 °C, agitation was set 150 rpm for the 96 h of cultivation period. 

Bacterial growth monitoring was carried out periodically by aseptically removing 0.5 mL aliquots and diluting them sequentially in one-quarter strength Ringer solutions (Lab M, Heywood, UK). For each sample, 0.5 mL was serially diluted in 10-fold steps to 10^−7^. Ringer solution was prepared by dissolving 1 tablet in 500 mL of deionised water in a 1-L flask. Cell viability was determined by serial dilutions onto Petri dishes containing nutrient agar. Each dilution was plated following the Miles and Misra method [37] and employing 20 µL of each dilution. Colonies were counted after overnight incubation at 37 °C and organismal concentration was expressed as Log Colony Forming Units/mL. All results were statistically analysed using Microsoft Excel and SPSS 25.

#### 2.1.4. Statistical Analysis

All cultivation parameters and yield presented were undertaken in triplicates. Results were statistically analysed by means of standard deviation, standard error and two-sided *t*-test using SPSS 25.

#### 2.1.5. Isolation and Purification of γ-PGA

For material isolation, the culture broth was withdrawn from the fermentation vessel and centrifuged at 17,000× *g* for 30 min to remove cells, by employing a ERMLE Z 300K centrifuge (Wehingen, Germany). The supernatant was poured into 3 volumes of cold ethanol and left overnight at 5 °C to precipitate. The resultant precipitate was collected by filtration over a 0.22 µm paper filter (Fischer Chemicals Ltd., Loughborough, UK). The precipitate was subsequently lyophilised (Alpha 1–4 LSC plus Christ Freeze Dryer, SciQuip Ltd. Bomere Heath, UK) with a 36 h run. The white/green/brown, dry powder was stored in a desiccator at room temperature until further use.

For further purification, the obtained dry powder was re-dissolved into deionised water and dialysed against deionised water within a cross flow system purchased from Repligen, US with a 20 cm MidiKros column of 30 KDa cut off from Repligen. The purified media was re-precipitated with 3 volumes of cold ethanol and left at 5 °C overnight. After precipitation the polymer was collected and lyophilised. 

#### 2.1.6. Characterisation of γ-PGA

The purified polymer isolated form was identified by FT-IR with Nicolet 380 FT-IR (Thermo Fisher Scientific Inc., Wilmington, DE, USA) with 32 scans and 4 cm^−1^ resolution. The measurements were over 100 scans and wave number range of 400–4000 cm^−1^. FT-IR for γ-PGA-HAp interaction was performed using the Perkin Elmer FT-IR System Spectrum GX equipment and software. The dry HAp powder (Plasma Biotal, Tideswell, UK) was placed on a clean stage, where scans were performed in the wavelength range of 500–2000 cm^−1^. Each spectrum was obtained based on an average of 10 scans. HAp disc (Plasma Biotal, Tideswell, UK) and loose HAp powder (Plasma Biotal, Tideswell, UK) references were recorded. Spectral scans were recorded of the exposed surface of the HAp discs after being treated. Treatment with HAp powder, was undertaken by placing 0.7 g (the equivalent weight of a HAp 20% porous disc) in 10 mL of 2% γ-PGA-Na (Table 1) and gently agitated in the formulation for 2 min before re-pelleting in a centrifuge for 2 min at 4000 rpm. The remaining γ-PGA was drawn out and the powder dried in a heat dryer for 1 h before a spectral scan was run.

The average molar mass and molar mass distributions of the polymers were determined by gel permeation chromatography (GPC) with a differential refractive index detector (Δn-2010 RI WGE Dr. Bures, Berlin, Germany) and a multiangle laser light scattering detector (DAWN EOS, Wyatt Technologies, Santa Barbara, CA, USA) in buffer (0.15M NaNO_3_, 0.01M EDTA, 0.02% NaN_3_ and pH = 6 adjusted with NaOH). The following columns were used: guard PSS SUPREMA 10 μm and PSS SUPREMA analytical Linear XL 10 μm (Polymer Standards Service, Mainz, Germany). Measurements with nominal flow rate of 0.5 mL/min, at 40 °C were performed. ASTRA 4 software (Wyatt Technologies, Santa Barbara, CA, USA) was used to evaluate the results. All samples were filtered through the 0.45 µm PES syringe filters (Graphic Controls, DIA-Nielsen, Düren, Germany) before measurements. The refractive index increment of commercial γ-PGA (dn/dc = 0.142 mL/g) was estimated in independent measurement in buffer using a SEC-3010 dn/dc WGE Dr. Bures (Berlin, Germany) differential refractive index detector.

To attain high quality diffraction data for γ-PGA samples, a PANanalytical Empyrean X-ray diffractometer was employed. For sample analysis, approximately 200 mg of pure γ-PGA powder was homogeneously flattened within the holder with the aid of a glass slide. Analysis conditions employed were: Scan Axis: Gonio, Start Position [°2Th.]: 5.0090, End Position [°2Th.]: 99.9870, Step Size [°2Th.]: 0.0130, Scan Step Time [s]: 8.6700, Irradiated Length [mm]: 15.00, Specimen Length [mm]: 10.00, Measurement Temperature [°C] 25.00, Generator Settings: 40 mA, 40 kV, Goniometer Radius [mm]: 240.00, Dist. Focus-Diverge. Slit [mm]: 100.00.

#### 2.1.7. Formulation of γ-PGA

The γ-PGA solutions were prepared using commercial and algal-derived γ-PGA powder from *Bacillus subtilis* natto (see Table 1). The molar mass of these γ-PGAs ranged from 4 to 3000 kDa. Solutions were made in different concentrations in the ranges of 0.25 to 4% (wt), with deionised water. Equal amounts of NaOH solutions in deionised water were prepared and added to the γ-PGA solutions to produce a soluble γ-PGA with pH ~ 7.00. Solutions using the sodium salt γ-PGA samples, with M of 66 kDa (hydrolysed from material obtained by Xi’an Zhongyum Biotechnology Co., Ltd., xi’an, China), 166 kDa (Nippon Poly-Glu Co., Ltd., Osaka, Japan), 520 kDa γ-PGA (CS Innovation LLC., Schenectady, NY, USA) and 440 kDa (YRSpec, Tianjin, China) were prepared using deionised water. 

#### 2.1.8. Artificial Demineralisation Monitored Using ISE

Synthetic HAp discs (20% porosity—obtained from Plasma Biotal (Tideswell, UK)) γ-PGA protected or not, were immersed in a 0.1M acetic acid solution. The demineralisation solution of 0.1M acetic acid was prepared and buffered to pH 4.0 with 1M Sodium Hydroxide (NaOH) using a calibrated pH meter (Mettler Toledo: inLab Expert Go-ISM).

HAp discs were immersed in 5 mL of γ-PGA solutions for 2 or 5 min. A sealed test tube was used and manually shaken to replicate the swishing motion of a mouthwash. The discs were then immersed in 50 mL of 0.1M acetic acid, pH 4, for 1 h at 37.0 (±1.0 °C)

#### 2.1.9. ISE or Real-Time Monitoring of Artificial Caries 

ISEs have been used to measure the rate of loss of calcium from enamel or HAP discs as a proxy for the rate of demineralisation as described by [38]. A Ca^2+^ ion selective electrode (ISE) was paired with a double-junction lithium acetate reference electrode. The electrodes were calibrated using a serial dilution technique with a solution of pH4 and at 37 (±1.0 °C) while using a magnetic stirrer. A calibration curve was obtained by plotting the logarithm of calcium activity in millimols against the ISE readings in millivolts. For ISE readings, Ion-Selective Electrode for calcium ion (ELIT 8041 PVC membrane), a reference electrode: single junction silver chloride (ELIT 001n) was mounted on a dual electrode head (ELIT 201) obtained from Nico2000 (London, UK). An ELIT software was used to record the data Nico2000 (London, UK).

## 3. Results

### 3.1. Biosynthesis of γ-PGA from Macro-Algae and Micro-Algae Waste Fraction

**Figure 2:**
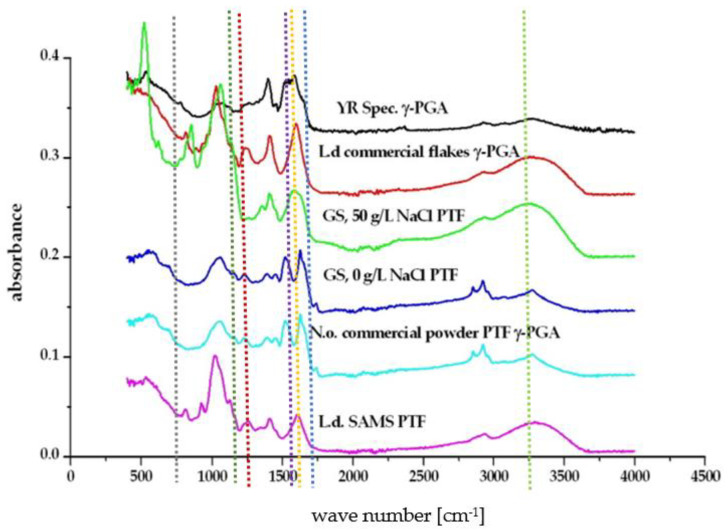
Fourier Transform Infra-Red spectra of γ-PGA obtained from different standard and complex waste substrates. The blue vertical line represents the C=O stretch, the orange line represents the Amide I N-H bend, the purple line represents the Amide II stretch, the red line represents the C=O symmetric stretch of γ-PGA in its sodium (1402 cm^−1^) or calcium (1412 cm^−1^) isoform, the dark green line represents the C-N stretch, the grey line represents the N-H bending, the light green line represents the O-H stretch.

The strong need for novel sustainable materials and products has significantly boosted algal agriculture. Herein, we tested the potential of algal substrate as a source of nutrients for the biosynthesis of value-added materials. FT-IR data [22] suggested that the brown macro-algae *Laminaria digitata* and the post-oil extracted micro-algae *Nanochloropsis oceanica* are suitable substrates for *Bacillus subtilis* natto biosynthesis of γ-PGA (Figure 2) [24]. 

**Figure 3:**
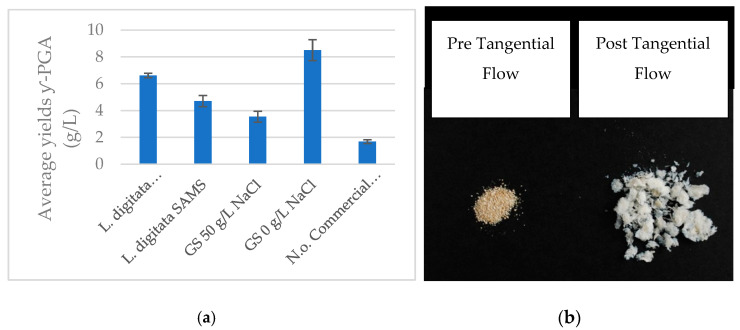
(**a**) Average yields of γ-PGA obtained post-tangential flow with supplemented Laminaria digitata substrate (in flakes from commercial supplier and in powder from Scottish Association for Marine Science), pre-extracted and supplemented Nanochloropsis oceanica media, standard GS media (50 g/L NaCl) and modified GS media (0 g/L NaCl). (**b**) Pre- and post-tangential flow of purified γ-PGA obtained from L.d. commercial substrate. n = 3. Error bars indicate standard error values.

Herein, *B. subtilis* natto cultivated on supplemented *L. digitata* flakes/powder and pre-extracted *N. oceanica* displayed significant biosynthesis of γ-PGA (Table 1, Figure 3). The data presented within Figure 3 suggested that variation in *Laminaria digitata* processing (commercial versus experimental treatments) significantly (*p* < 0.001) impacted polymeric yields after tangential flow purification (*L. digitata* commercial vs. *L. digitata* SAMS within Figure 3). Post-tangential flow yields of γ-PGA obtained from commercial *L. digitata* flakes were significantly higher compared to those obtained with standard GS media (*p* = 0.003). Differently, yields of γ-PGA obtained from commercial *L. digitata* flakes were approximately 22.5% lower compared to those obtained with standard GS media at 0 g/L NaCl. This difference was found to be significant (*p* < 0.001). The yields of γ-PGA from pre-extracted, supplemented *N. oceanica* were significantly lower (*p* = 0.003) than those obtained with either supplemented macro-algae media or standard GS media.

### 3.2. Physico-Chemical Characteristics of γ-PGA Synthesised from Macro-Algae and Micro-Algae Waste Fraction

As presented in Table 2, the molar mass of γ-PGA produced from *L. digitata* commercial flakes was lower compared to that obtained from SAMS monthly collected *L. digitata* samples. The molar mass of γ-PGA produced from *L. digitata* samples remained relatively constant across the months assessed. Surprisingly, the molar mass of SAMS *L. digitata* samples was comparable to that obtained with standard GS medium at 0 g/L NaCl. The molar mass of both standard GS media and GS media 0 g/L NaCl were significantly higher than those reported in literature for members of *Bacillus* sp. The molar mass for PTF *N. oceanica* was extremely low compared to other γ-PGAs isolated.

### 3.3. The Impact of γ-PGA Concentrations on HAp Demineralisation

**Figure 4:**
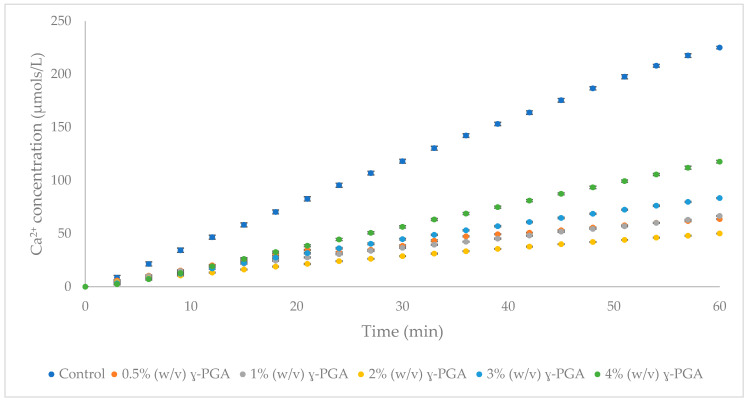
ISE study on the Ca^2+^ dissolution from HAp discs (20% porous) after being treated for 2 min with analogous γ-PGA solutions at different concentrations. n = 3. Error bars indicate standard error values.

Ion selective electrode investigations are a generally accepted model employed to assess the demineralisation of human enamel in response to varying treatments [38]. The rate of HAp dissolution with increasing concentrations of γ-PGA was assessed with the aid of porous HAp discs. To this end, 0.5 to 4% (*w*/*v*) formulations of γ-PGA, with molar mass 66 kDa, were investigated and the results of this investigation are summarised in Figure 4.

All γ-PGA formulations tested imparted significant demineralisation inhibition on the HAp discs (Figure 4), with the greatest protection between 1 and 2% (*w*/*v*). The γ-PGA protection, from high to low, was observed as follows: 1% (*w*/*v*) > 2% (*w*/*v*) > 0.5% (*w*/*v*) > 3% (*w*/*v*) > 4% (*w*/*v*) γ-PGA formulations. 

### 3.4. Comparison of γ-PGA Average Molar Mass on HAp Protection

**Figure 5:**
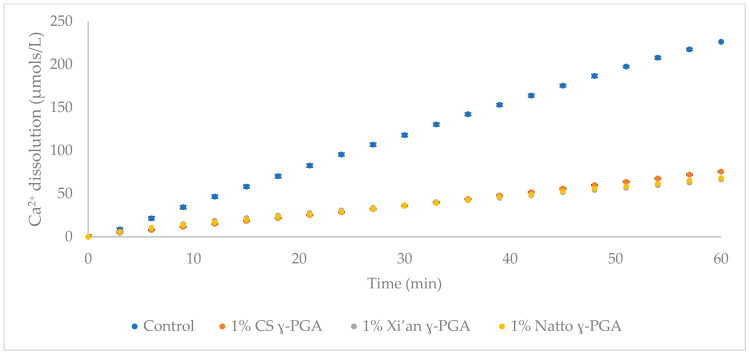
Linear regression analysis of the ISE pilot study result showing the Ca^2+^ activity from HAp disc (20% porous) protected by different commercial γ-PGA at 1% (*w*/*v*) with disc immersion for 2 min. n = 3. Error bars indicate standard error values.

Having established that the best HAp disc protection occurred with formulations presenting γ-PGA at 1% (*w*/*v*), commercial γ-PGAs with varying molar masses were assessed. The results of this investigation are schematically summarised in Figure 5.

There was little difference between the γ-PGA molecular mass tested and demineralisation inhibition (Figure 5). To assess whether this behaviour is concentration specific, or observed across all γ-PGA containing samples, 0.1 and 0.25% (*w*/*v*) formulations of γ-PGA were tested on HAp discs. 

**Figure 6:**
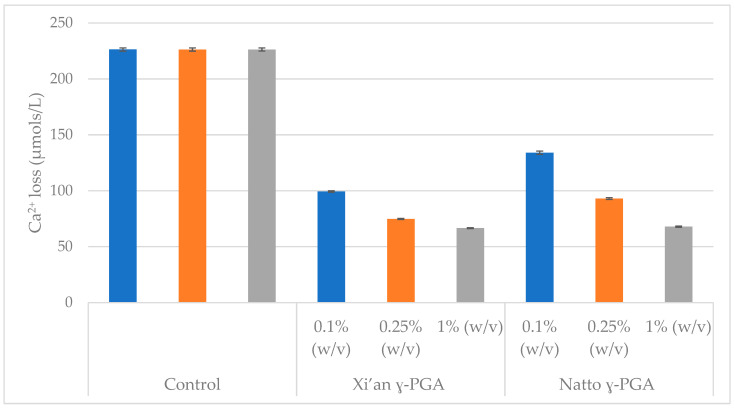
Comparison of the ISE pilot study result showing the Ca^2+^ activity from HAp disc (20% porous) protected by different commercial γ-PGAs at 0.1, 0.25 and 1% (*w*/*v*). n = 3. Error bars indicate standard error values.

The overall optimum of HAp protection was achieved with 1% (*w*/*v*) γ-PGA (Figure 6). Significant variation was observed when both γ-PGA molar masses and its concentrations were compared. In this respect, variation in molar mass provided significant variation (*p* < 0.05). Further, different concentrations also presented significant variation in HAp protection (*p* = 0.028) (Two-way ANOVA).

From Figure 6 it appeared that better protection was achieved with 1% (*w*/*v*) formulations compared to 0.25% (*w*/*v*) formulations. Hence, we came to the conclusion that, regardless of chemical properties (Table 2), 1% (*w*/*v*) γ-PGA formulations were the most suitable in protecting HAp from dissolution across the range tested (0.1, 0.25, 0.5, 1, 2, 3 and 4% (*w*/*v*)). 

**Figure 7:**
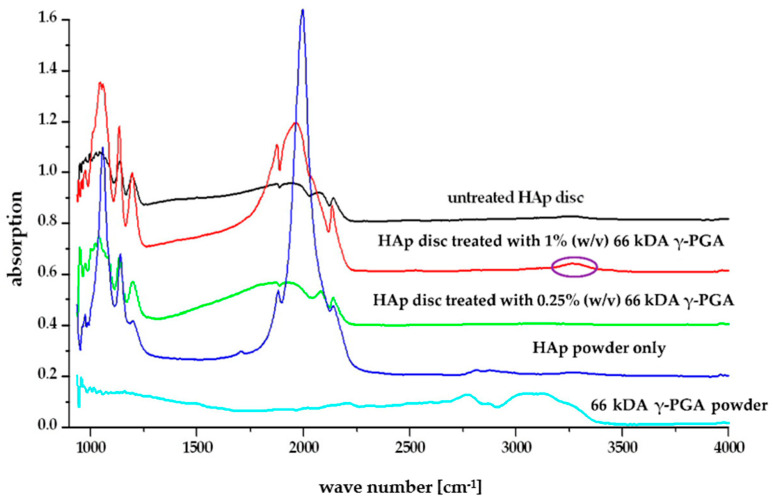
FT-IR profiles of HAp disc before and after 1 h acidic challenge in the presence of γ-PGA. Interaction peak, between γ-PGA and HAp disc, circled in purple.

To further investigate the association between γ-PGA and teeth enamel, FT-IR assessment was carried out for 0.25% (*w*/*v*) and 1% (*w*/*v*) formulations (see Figure 7). A small peak at the 1500–1600 cm^−1^ region of the spectra was visible on all HAp discs treated with both 0.25% (*w*/*v*) and 1% (*w*/*v*) γ-PGA after being immersed in 0.1M acetic acid for 1 h.

### 3.5. Enamel Protection from Micro- and Macro-Algal Produced γ-PGA

**Figure 8:**
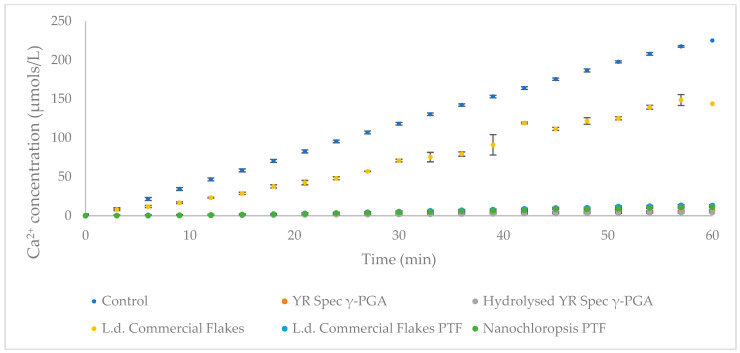
Protective effect of 1% (*w*/*v*) γ-PGA formulations obtained from micro- and macro-algal substrate towards HAp discs. The γ-PGA formulations tested were comprised of water only (control), commercial -440 KDa- γ-PGA (YR Spec γ-PGA), Commercial γ-PGA hydrolytically degraded (Hydrolysed YR Spec γ-PGA), γ-PGA produced from algal biomass raw precipitate, before tangential flow (L.d. Commercial Flakes), γ-PGA produced from algal biomass after tangential flow (L.d. Commercial Flakes PTF), γ-PGA produced from pre-solvent extracted, waste, micro-algal biomass fraction after tangential flow (N.o Commercial Powder PTF). A detailed summary of the cultivation media components (where applicable) can be found within Table 1. All HAp discs were immersed in the formulation for 5 min. n = 3. Error bars indicate standard error values.

Cost effective biosynthesis of raw materials is fundamental in product development. For this reason, sustainable and efficient synthesis of γ-PGA is necessary. To this end micro- and macro-algae substrates were employed for the synthesis of γ-PGA. Figure 8 summarises the HAp protection capability of γ-PGA synthesised from micro- and macro- algal substrates.

The data again showed that all of the 1% (*w*/*v*) formulations tested demonstrated HAp protection, compared to control. The formulation which displayed the least protection was L.d. Commercial Flakes. All the other formulations displayed similar demineralisation inhibition.

## 4. Discussion

### 4.1. Variation in γ-PGA Yields and Physico-Chemical Properties with Micro- and Macro-Algal Substrate

Cost effective biosynthesis of ultra-pure γ-PGA is fundamental for its use in the biomedical field. To this end, we employed wild-cultivated macro-algae and solvent extracted micro-algae and tested their potential as substrates for γ-PGA biosynthesis. Our data suggested that both supplemented macro-algae and pre-extracted, supplemented micro-algae allowed for γ-PGA biosynthesis. Conventionally, γ-PGA is synthesised by members of the *Bacillus* species with average yields ranging from 4 g/L to 35 g/L [39,40,41,42,43,44,45]. Depending upon the nature of the substrate, yields reported can be of raw precipitates. In our study we reported yields of γ-PGA raw precipitate obtained from standard GS media of 9.8 g/L for 50 g/L NaCl and 14.5 g/L for 0 g/L NaCl, with 3.5 and 8.5 g/L from tangential flow purified GS media and GS media at 0 g/L NaCl. For complex waste media we reported raw yields at 10.3 g/L for *L. digitata* collected by SAMS and at 8.8 g/L for commercially available *L. digitata* flakes (data not shown). As summarised in Figure 3, purified *L. digitata* yielded 4.8 g/L and 6.6 g/L γ-PGA for samples collected by SAMS and commercial samples, respectively. These results were in line with the only other report of γ-PGA production from macro-algae (*Ulva* spp.) by Kim et al., (2019) [32]. Therein Kim et al., (2019) [32] reported a yield of 6.29 g/L of purified γ-PGA, in line with 6.6 and 4.78 g/L reported herein. The significant variation in yields observed can be explained by the variation in micronutrient content present within the algal substrate. It has been widely reported that the concentrations of Ca^2+^, Zn^2+^, Co^2+^, Zn^2+^, Mg^2+^, Na^2+^, Fe^2+^ and Mn^2+^ impact the yields, molar mass and D/L ratios of the resulting material [32,39,40,43,44,46,47,48,49]. The variation in concentration of these minerals could be greatly affected by the location of cultivation or by the pre-treatment method (sterilisation) commercial *L. digitata* could have incurred before packaging and distribution [50]. In this case, complex carbohydrates and proteinaceous material would have been mostly degraded into simple sugars and amino acids, respectively. These building blocks could have stimulated growth of *B. subtilis* natto more, compared to the SAMS *L. digitata* substrate. To the best of our knowledge, this is the first report of successful γ-PGA production from micro-algae. Herein, we reported the synthesis of 1.68 g/L of γ-PGA from supplemented, solvent extracted *Nanochloropsis oceanica*. 

Although the yields obtained from pre-extracted micro-algae were relatively low, this cheap waste source could be particularly interesting for its valorisation, especially because the polymer produced presents relatively low molar mass of 4.6 kDa, often difficult to obtain with conventional substrates. Furthermore, another advantage of micro-algae-based media is the greater biomass consistency obtained, when cultivated in photobioreactors, compared to macro-algae, conventionally cultivated in ocean plots. In that respect, molar mass obtained with standard GS media and GS media without NaCl were 2700 kDa and 3700 kDa, respectively, which are significantly higher than those reported in literature for members of the *Bacillus* sp., which commonly range from 100 kDa to 2100 kDa [34,39,40,41,42,44,45], but analogous to the 2575 kDa reported by Bhat et al., (2013) [51] for *Bacillus subtilis* natto cultivated in standard GS media (50 g/L NaCl). Surprisingly, the variation in molar mass between commercial and SAMS samples of *L. digitata* observed was 16-fold. In fact, all samples of *L. digitata* collected by SAMS between 07.04.2021 and 01.07.2021 led to γ-PGA masses above 3000 kDa, compared to 184 kDa obtained from commercial samples. This result further supported the hypothesis previously formulated, for which it is suggested that variation in concentrations of Ca^2+^, Zn^2+^, Co^2+^, Zn^2+^, Mg^2+^, Na^2+^, Fe^2+^ and Mn^2+^ impact the yields, molar mass and D/L ratios of the resulting material [32,40,41,45,47,48,49]. 

### 4.2. Affinity and Protection of γ-PGA towards HAp 

Alternative substrate engineering for the biosynthesis of γ-PGA, and increased understanding of its ability to maintain the integrity of dental enamel at low pH conditions, provides novel insights into the behaviour and physico-chemical interaction of this biomaterial. To assess the proposed protective effect of γ-PGA towards enamel, we used a synthetic enamel-like model system (20% porous calcium HAp discs), that has been used in many artificial caries studies [39,52,53]. Although these analogous materials do not have the carbonated HAp of enamel, chemical differences are small, However, structurally they are very different. Human tooth enamel consists of non-homogenous carbonated hydroxyapatite and contains ionic substitutions within the crystal lattice [8]. A pure calcium HAp has a higher solubility product than a non-homogenous carbonated HAp. Human enamel has a typical porosity of 1%. During acidic challenge, disassociated protons penetrate these pores causing dissolution of the tightly packed, parallel HAp enamel crystallites in the subsurface layers [20]. The synthetic HAp discs used here have a greater porosity, 20%, allowing for a much greater amount of demineralisation solution to penetrate the structure of the disc. The porous structure is not uniform throughout the disc and may interconnect throughout the entire structure [20]. This leads to greater surface contact of the protons with the HAp crystals, increasing the rate of Ca^2+^ dissolution released from the discs [20]. Untreated HAp discs released Ca^2+^ at a rate of 3 µmol/L/min in acetic acid of pH4, as extrapolated from Figure 4. 

However, when 20% HAp discs were immersed in 0.1, 0.25, 0.5, 1, 2, 3 and 4% (*w*/*v*) formulations of γ-PGA for 2 min, Ca^2+^ dissolution could be reduced below 2 µmol/L/min, regardless of the concentration of γ-PGA. Figure 4 suggested that the lowest Ca^2+^ dissolution rate was observed with 1% (*w*/*v*) γ-PGA formulations. This result was not surprising, as lower γ-PGA concentrations struggle to effectively coat the surface of the disc, whereas higher concentrations are hypothesised to display greater chain to chain interaction, bridged by Ca^2+^ ions, rather than chain to HAp interaction. Although, chain length and enantiomeric profile are known to play a crucial role in polymeric interactions [54], this did not appear to be the case for commercial γ-PGA formulations at 1% (*w*/*v*) of different molar mass (Figure 5). Nonetheless, when the concentration of γ-PGA formulations was reduced, a distinct separation in protective effect was noticed. In fact, Figure 6 suggested that a significant variation in protective effect was observed with variation in polymeric molar mass. Our data suggested that at 0.25% (*w*/*v*) γ-PGA concentrations, higher molar mass provided the lowest Ca^2+^ dissolution, whereas at 0.1% (*w*/*v*), the lowest molar mass material displayed the greatest protection. This apparent dichotomy could be explained as follows: at 0.25% (*w*/*v*), the greater the molar mass of the material, more surface area is covered and lower demineralisation observed. In contrast, with lower molar mass polymers, enantiomeric properties significantly modulate the attachment of the material and ultimately lower Ca^2+^ dissolution. As discussed earlier, previous research suggests that the L-glutamic acid component of statherin is responsible for its attachment to the enamel [12,13], and, thus, it is possible that samples presenting a higher content of l-glutamic acid units had better affinity compared to samples that presented higher d-glutamic acid units. When the concentration of γ-PGA was reduced to 0.25% (*w*/*v*), the variation in protection was not limited to molar mass but could also possibly be affected by D/L enantiomeric distribution. Both the molar mass and the enantiomeric ratio are assumed to affect the protection as a consequence of γ-PGA binding to HAp disc. This multivariate behaviour can also be complicated by the presence of ions associated with the γ-PGA. A similar molar mass-protection pattern was also observed with 0.1 *w*/*v*% γ-PGA, but, in this case, 520 kDa and 200–400 kDa γ-PGA displayed analogous protection with 66 kDa γ-PGA displaying higher protection. These chemico-physical aspects have to be further investigated.

At both 0.25% (*w*/*v*) and 1% (*w*/*v*) concentration, the FT-IR data suggested that γ-PGA was chemically bound to HAp as novel peaks were observed at 1580 and 1620 cm^−1^ (Figure 7). These peaks were characteristic of γ-PGA and represented the α- and γ- amide linkages in the γ-PGA and were contributed to by -C=O stretching and -N-H bending modes [24]. These peaks were not visible in the spectra for untreated HAp powder; therefore, their presence on the treated HAp spectra indicated that γ-PGA had bound to the surface of the HAp [20]. The binding of γ-PGA to HAp has been suggested to occur due to the high affinity of the α-carboxyl groups of the residual side chains to calcium, forming stable ionic complexes via chelating ligands [55]. As the proposed HAp-γ-PGA and HAp-statherin binding mechanisms are suggested to be analogous, the observed reduction in Ca^2+^ dissolution could be a direct consequence of the shielding behaviour carried out by both γ-PGA and statherin. The shielding proposed effectively reduced the interaction between the acidic environment (H^+^) and the HAp layer thereby reducing the concentration of soluble products of calcium hydroxyapatite, and ultimately preventing bioerosion. Although very weak, small peaks in this region were also visible on all spectra of the HAp discs after they had been treated with γ-PGA at different concentration and molar mass (Figure 7) following constant acidic challenge. This indicated that the concentration and the average molar mass did not affect the binding ability of γ-PGA to the HAp surface and that binding remained after a constant acidic challenge at pH, further supporting the shielding behaviour of γ-PGA.

### 4.3. HAp Protection through Micro- and Macro-Algal Produced γ-PGA 

The γ-PGA synthesised from micro- and macro-algal substrate was further assessed by protection capability for HAp protection. In this case, five 1% (*w*/*v*) γ-PGA formulations were prepared and tested. The data summarised within Figure 8 suggested that, when HAp discs are exposed to γ-PGA formulations for 5 min, all tangential flow-purified polymers displayed >92% reduction in Ca^2+^ dissolution. Differently from the other formulations tested, sample ‘L.d commercial’ only displayed 31% reduction in Ca^2+^ dissolution, compared to the control. This variation in protection was attributed to the fact that the material produced had not undergone tangential flow purification. Being produced from a carbohydrate and protein rich substrate, non-γ-PGA-selective ethanol precipitation led to an inhomogeneous material. This hypothesis was greatly supported by the GPC investigations which suggested a dispersity index of 13, compared to the 3.2 dispersity index obtained with the tangential flow-purified sample (Table 2). Within this pre-purification sample, it was very likely that γ-PGA chains’ available pendant carboxylic acids excessively interacted with other molecules and proteins, compared to HAp disc. 

## 5. Conclusions

To conclude, we successfully demonstrated the biosynthesis of γ-PGA from cheap, complex sources (namely micro-algae and macro-algae) and its ability to protect enamel dissolution. The yields of pure polymer obtained from these sources was found to be higher compared to the costly GS media. However, that was not the case when the NaCl concentration of the GS media was reduced from 50 g/L to 0 g/L, in which case higher yields were obtained with the standard media. Interestingly, γ-PGA obtained by SAMS *Laminaria digitata* presented very high molar mass of 3000 kDa, analogous to those obtained with both GS media and GS media 0 g/L NaCl. 

Investigations into γ-PGA-HAp interaction suggested that 1% (*w*/*v*) γ-PGA formulations presented the greatest HAp protecting properties. Although the molar mass did not appear to affect HAp protection capabilities at 1% (*w*/*v*), we showed that there was significant variation in HAp protection at lower γ-PGA concentration (0.25 and 0.1% (*w*/*v*)) (*p* = 0.028). The variation in such protective effect was attributed to both molar mass and, potentially, to the enantiomeric properties of the polymers. To further assess the effect of enantiomeric properties towards enamel attachment, further investigation will focus on the D- and L-glutamic acid residue and their effects towards HAp binding.

FT-IR analysis suggested that there was physical interaction between γ-PGA and HAp for all formulations tested. This interaction was evidenced by a small peak in the 1500–1600 cm^−1^ region. We aim to further confirm and visualise such interaction through micro-CT and other imaging techniques to establish the bonding and its efficiency over time. 

Further, we have shown that by treating HAp discs with purified, micro- and macro-algae-derived γ-PGA significant reduction in enamel dissolution was achieved. The combination of purified polymers, alongside a five minute exposure of HAp discs to the γ-PGA formulation displayed a reduction in Ca^2+^ dissolution from 227µmol/L (control) to 11.4 and 14.1 µmol/L for N.o commercial PTF and L.d. commercial PTF, respectively. 

## Figures and Tables

**Table 1:** Summary of γ-PGA sample codes and relevant description.

Code	Description	Substrate
Xi’an γ-PGA	Commercial γ-PGA, hydrolysed to 66 kDa	Not specified
Natto γ-PGA	166 kDa commercial γ-PGA	Not specified
CS γ-PGA	520 kDa commercial γ-PGA	Not specified
YR Spec γ-PGA	440 kDa commercial γ-PGA	Not specified
Hydrolysed YR spec γ-PGA	102 kDa commercial hydrolysed γ-PGA	Not specified
GS, 0 g/L NaCl PTF	Modified commercial substrate	20 g/L L-glut, 50 g/L sucrose, 2.7 g/L KH_2_PO_4_, 4.2 g/L Na_2_HPO_4_, 0 g/L NaCl, 5 g/L MgSO_4_ ·7H_2_O, 1 mL/L of Murashige-Skoog vitamin solution
GS, 50 g/L NaCl PTF	Commercial substrate	20 g/L L-glut, 50 g/L sucrose, 2.7 g/L KH_2_PO_4_, 4.2 g/L Na_2_HPO_4_, 50 g/L NaCl, 5 g/L MgSO_4_ ·7H_2_O, 1 mL/L of Murashige-Skoog vitamin solution
L.d. Commercial flakes	Flakes from Cornish Seaweed	High shear mixing 3000 rpm for 3 min, 10 g/L L-glut, 25 g/L sucrose
L.d. Commercial flakes PTF		
L.d. SAMS PTF	Powder from Scottish Association for Marine Science ltd.	
N.o. Commercial Powder PTF	Powder from Algaecytes Ltd.	Ethanol extraction for 24 h, 10 g/L L-glut, 25 g/L sucrose

**Table 2:** Physico-chemical properties of commercial γ-PGA and γ-PGA synthesised from pre-extracted supplemented micro-algae media and pre-treated supplemented macro-algae media. A detailed summary of the cultivation media components (where applicable) can be found within Table 1. L.d.—*Laminaria digitata*, PTF: post-tangential flow purification.

Production Method	M_n_ [g/mol]	M_w_ [g/mol]	M_w_/M_n_	XRD
Xi’an γ-PGA	N.S.	66,000	Not Specified	Amorphous
Natto γ-PGA	47,800	166,000	3.5	Crystalline
CS γ-PGA	N.S.	520,000	Not Specified	Amorphous
Commercial γ-PGA	250,000	440,000	1.8	Amorphous
Hydrolysed commercial γ-PGA	33,200	102,000	3.1	Amorphous
GS, 0 g/L NaCl PTF	3,320,000	3,700,000	1.1	Amorphous
GS, 50 g/L NaCl PTF	1,810,000	2,700,000	1.5	Crystalline
L.d. Commercial flakes	10,900	145,000	13.3	Amorphous
L.d. Commercial flakes PTF	59,000	183,000	3.1	Amorphous
L.d. SAMS PTF	1,760,000	2,700,000	1.5	Amorphous
N.o. Commercial Powder PTF	1500	4600	3.1	Amorphous
